# Neuroprotective efficacy of thymoquinone against amyloid beta-induced neurotoxicity in human induced pluripotent stem cell-derived cholinergic neurons

**DOI:** 10.1016/j.bbrep.2018.12.005

**Published:** 2019-01-03

**Authors:** A.H. Alhibshi, A. Odawara, I. Suzuki

**Affiliations:** a Department of Neuroscience, Institute of Research and Medical Consultations (IRMC), Imam Abdulrahman Bin Faisal University, P.O.Box 1982, Dammam 31441, Saudi Arabia; b Department of Electronics, Graduate School of Engineering, Tohoku Institute of Technology, 35-1 Yagiyama Kasumicho, Taihaku-ku, Sendai, Miyagi 192-0982, Japan

**Keywords:** Thymoquinone, Amyloid beta, Alzheimer's disease, Human induced pluripotent stem cell-derived cholinergic neurons, Oxidative stress

## Abstract

The natural antioxidant Thymoquinone (TQ) is the most abundant ingredient in the curative plant Nigella sativa seed's oil. An extensive number of studies have revealed that TQ is the most active and most responsible component for the plant's pharmacological properties. It has been documented in several studies that TQ has a wide range of protective activities and many neuropharmacological attributes. Amyloid beta (Aβ) is the major role player peptide in the progression of Alzheimer's disease (AD). Our current study has been implemented to explore the protective possibilities of TQ on Aβ_1–42_ -induced neurotoxicity. To test TQ's effect we used cultured human induced pluripotent stem cell (hiPSC)-derived cholinergic neurons. The obtained results showed that Aβ_1–42_ caused cell death and apoptosis, which was efficiently attenuated by the co-treatment of TQ. Moreover, TQ restored the decrease in the intracellular antioxidant enzyme glutathione levels and inhibited the generation of reactive oxygen species induced by Aβ_1–42_. Furthermore, using the fluorescent dye FM1–43 we demonstrated that TQ was able to reduce synaptic toxicity caused by Aβ_1–42_. Thus, the findings of our study suggest that TQ holds a neuroprotective potential and could be a promising therapeutic agent to reduce the risk of developing AD and other disorders of the central nervous system.

## Highlights

• TQ protected hiPSC-derived cholinergic neurons against Aβ_1–42_ induced apoptosis.
• TQ restored reduced Glutathione level in hiPSC-derived cholinergic neurons.
• TQ protected hiPSC-derived cholinergic neurons against ROS generation induced by Aβ_1–42_.
• TQ attenuated Aβ_1–42_ – induced synaptic toxicity.

## 1 Introduction

As a neurodegenerative disorder, Alzheimer's disease (AD) slowly destroys memory and cognitive function and is known to be the most prevalent cause of dementia in the elderly [1]. In AD patient brain, there are two distinctive histopathological abnormalities: (1) the intracellular neurofibrillary tangles consisting of hyper phosphorylated tau protein, and (2) the extracellularly formed plaques composed of amyloid beta (Aβ) peptide. The cholinergic neurons system has been a major focus in neurodegenerative research and aging for many years since it has a strong correlation with AD [2]. Among the earliest well-established pathological events in AD, is the impairment in function and loss of basal forebrain cholinergic neurons and their cortical cholinergic innervation that strengthen the hippocampus and the neocortex [3], [4]. This process of neurodegeneration is triggered by the accumulation of Aβ peptide [5], which have also been hypothesized to induce neurodegenerative changes at cholinergic terminals [6]. Additionally, other studies revealed that oligomeric Aβ induces cell death [7], encourages apoptosis by physically piercing the cell membrane, causes neurotoxic cascade and neurodegeneration that leads to AD [8], [9].

Oxidative stress occurs in the early phase of AD and is known to play an essential role in its pathology and development in relation to the presence of Aβ [10]. It is an important feature in AD marked by overproduction of reactive oxygen species (ROS), oxidation in neuronal lipids, proteins, DNA, and RNA leading to the dysfunction and loss of neurons [11]. As previously reported by several studies, AD brain suffers from a significant low content of antioxidant enzymes, which leads the brain to be more susceptible to toxic effects induced by Aβ [12]. Thus, antioxidants have been considered for a long time as an approach to slow down AD progression.

In recent years the herbal medicinal plant Nigella sativa has been actively investigated for its established historical and religion-based remedy for a wide range of health problems and therefore is gaining worldwide attention [13]. Reports have described *Nigella sativa* as possessing many therapeutic effects, including anti-inflammatory, antitumor, antimicrobial and immune potentiation in addition to antioxidant and neuroprotective effects [14], [15], [16]. Maintaining cell health should be considered as one of the most important strategies to prevent damage from oxidative stress, especially in areas vulnerable to oxidative stress such as the brain and that could be achieved by consuming nutrients rich in antioxidants, such as *Nigella sativa* [17].

The natural antioxidant thymoquinone (TQ) is most the bioactive ingredient of the volatile oil of Nigella sativa seeds. TQ seems promising due to its numerous biological properties, which include antioxidant, anti-inflammatory, and anticancer attributes [18], [19] that might be useful in the management of AD. It was demonstrated that in primary rat cortical neurons TQ has a protective role against ethanol-induced neuronal apoptosis [20]. TQ was additionally reported to reduce peroxidation levels, enhance enzymatic and non-enzymatic antioxidants activities in rats’ brain tissue [21], and protects against cytotoxic agents via attenuation of oxidative stress in PC12 cells [22]. Taking into consideration the rising attention in using herbal medicine for the treatment of chronic disorders, the neuroprotective possibilities of TQ seems to be hopeful in the management of neurodegenerative disorders.

Thus, in the current study, we evaluated the effect of TQ and Aβ_1–42_ on cell viability, caspase 3/7 activities, glutathione (GSH) level, ROS generation, and synaptic activity in human induced pluripotent stem cell (hiPSC)-derived cholinergic neurons.

## 2 Materials and methods

### 2.1 Cell culture

Human iPSC-derived cholinergic neurons cell line was obtained from (ReproCell, RCESDA001). 96 micro-well tissue culture plates were coated with poly-D-lysine for 2 h and then treated with (ReproCell) coating solution overnight. Cells were cultured at a density of 3.0 × 10^4^ cell/well and were grown at 37 °C in a humid atmosphere of 5% CO_2_ and 95% air.

### 2.2 Reagents and treatment

Amyloid β-protein 1–42 (Aβ_1–42_) was obtained from Peptide Institute Inc., prepared at 1 mM in dimethyl sulphoxide (DMSO, Wako) and stored at − 20 °C. A solution of Thymoquinone (TQ) obtained from (Sigma-Aldrich) was freshly prepared on the day of use at 10 mM in DMSO and final concentration was diluted in culture medium.

The concentration of TQ in this study was determined based on a previously established does curve [23] and the optimal concentration of TQ was selected based on cell viability. When TQ was applied in different concentrations simultaneously with Aβ_1–42_, it resulted in a striking improvement in cell survival, in a dose-dependent manner; and the maximal rescue occurred at a dose of 100 nM.

Additionally, it was previously established [24] that the treatment of human iPSC-derived neurons with Aβ_1–42_ (5 μM) resulted in neuronal toxicity when applied to cultures for 48 h. Therefore, in our current study, hiPSC-derived cholinergic neurons were treated with Aβ_1–42_ (5 μM) and TQ (100 nM). Cultures were treated on day 13 DIV.

### 2.3 Measurement of cell viability

The protective effect of TQ on cell viability of hiPSC-derived cholinergic neurons was determined by measuring ATP amount which is relevant to the number of live cells in culture using (CellTiter-Glo) assay from Promega. Cultures were treated with Aβ_1–42_ (5 μM) with or without TQ (100 nM) for 48 h. Cell viability was assessed according to the manufacturer's instructions. Luminescence signals were measured using (TECAN) microplate reader.

### 2.4 Measurement of caspase-3 and -7 activities

Using the Caspase-Glo 3/7 assay (Promega), the effect of TQ and Aβ_1–42_ on apoptosis was investigated by measuring Caspase 3 and 7 activities. Aβ_1–42_ (5 μM) was added to hiPSC-derived cholinergic neurons alone or simultaneously with TQ (100 nM) for 48 h. At the end of the incubation time, assay reagents were prepared according to the manufacturer's instructions and applied to the cells. After 1 h incubation at room temperature, a microplate reader (TECAN) was used to measure luminescence signals.

### 2.5 Measurement of antioxidant enzyme glutathione

Oxidative stress was assessed through measurement of the glutathione (GSH) using the GSH-Glo™ Glutathione assay (Promega). Seeded cells were treated with Aβ_1–42_ (5 μM) with or without TQ (100 nM) for 48 h. The assay was performed according to the manufacturer's instructions. After 48 h of the treatment, the first prepared reagent was applied to the cells and incubated for 30 min at room temperature. Then, the second prepared reagent of the assay was applied to the cells for 15 min at room temperature. A microplate reader (TECAN) was used to measure luminescence signals.

### 2.6 Measurement of intracellular ROS

Intracellular ROS level was determined by ROS™ H_2_O_2_ assay (Promega). The assay determines ROS level by measuring hydrogen peroxide (H_2_O_2_) concentration. Cultures were treated for 48 h with Aβ_1–42_ (5 μM) with or without TQ (100 nM). After 42 h, the first prepared reagent of the assay, was applied to the cells and the incubation continued for 6 h according to the manufacturer's instructions. Then, the second prepared reagent of the assay was applied to the cells for 20 min at room temperature. Using a microplate reader (TECAN) luminescence signals were measured.

### 2.7 Measurement of synaptic vesicles recycling activity (FM1–43 assay)

To assess the effect of Aβ_1–42_ and TQ on synaptic vesicles recycling, we used the fluorescent dye FM1–43 (Molecular Probes) that measures synaptic vesicles recycling. hiPSC-derived cholinergic neurons were treated with Aβ_1–42_ (5 μM) only or simultaneously with TQ (100 nM) for 48 h on culture day 13. On the final day of treatment, the culture medium was removed, and the neurons were incubated with 1 μg/ml artificial cerebrospinal fluid (ACSF)/FM1–43 solution for 5 min at 37 °C. Ice-cold phosphate buffered saline (PBS) was added to wash the cells three times and then for cell suspension. The excitation wavelength of FM 1–43 fluorescence intake was measured at 480 nm and the emission wavelength at 612 nm with (TECAN) microplate reader.

### 2.8 Statistical analysis

All data reported are expressed as mean ± SEM. Statistical significance of the results was calculated using one-way ANOVA followed by the Holm-Bonferroni method. The changes in parameters induced by all externally applied chemicals were quantified as a percentage of baseline values.

## 3 Results

### 3.1 Effect of Aβ_1–42_ and TQ on the survival of hiPSC-derived cholinergic neurons

**Fig. 1:**
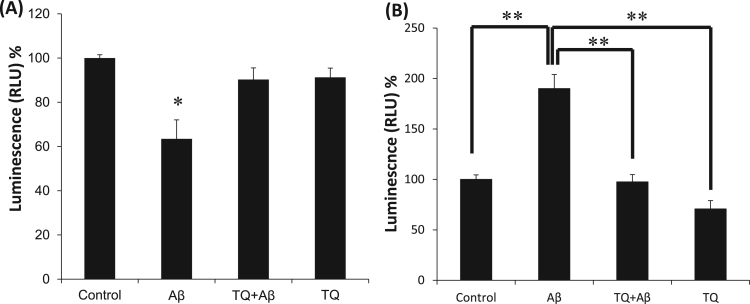
Effect of TQ and Aβ_1–42_ on cell viability and apoptosis in hiPSC-derived cholinergic neurons. (A) The group treated with Aβ_1–42_ (5 μM) had a significant 36.5% loss in their viability. In the group treated with Aβ_1–42_ and TQ (100 nM), TQ prevented Aβ_1–42_ induced cell death by restoring the cell viability to 90%. Cultures were treated for 48 h and viability was assessed with Cell Titer – Glo assay. (*P < 0.05 vs. control) n = 4. (B) Aβ_1–42_ (5 μM) treated group had a significant 90% increase in caspase 3/7 activities comparing to controls. However, simultaneous treatment with TQ (100 nM) abolished the activities to control levels significantly. Cultures were treated for 48 h and activities of caspase 3 and 7 were measured with Caspase-Glo 3/7 assay. (**P < 0.01: the group exposed to Aβ_1–42_ alone vs. control, vs. TQ+ Aβ_1–42_ group, vs. TQ alone), n = 5. Values shown are the mean percent luminescence (where 100% = luminescence in control hiPSC-derived cholinergic neurons), ± Standard Error of the Mean (SEM). Data were analyzed by one-way ANOVA, followed by the Holm-Bonferroni method.

The cytotoxicity of Aβ_1–42_ and the protective effect of TQ were evaluated using the CellTiter-Glo assay. Fig. 1A shows the effect of Aβ_1–42_ (5 μM) with or without TQ (100 nM) on hiPSC-derived cholinergic neurons viability. Treatment with Aβ_1–42_ for 48 h significantly decreased cell viability to 63.5% as compared to the control group (*P < 0.05). However, co-treating the cells with TQ (100 nM) prevented Aβ_1–42_ induced loss and protected the cells by restoring viability to %90.

### 3.2 Effect of Aβ_1–42_ and TQ on caspase 3/7 activities

As illustrated in Fig. 1B, treatment of hiPSC-derived cholinergic neurons with Aβ_1–42_ (5 μM) induced about 90% increase in the caspase 3/7 activities (^**^P < 0.01). However, TQ (100 nM) co-treatment restored caspase 3/7 activities to control sample level (^**^P < 0.01).

### 3.3 Effect of Aβ_1–42_ and TQ on level of antioxidant enzyme GSH

**Fig. 2:**
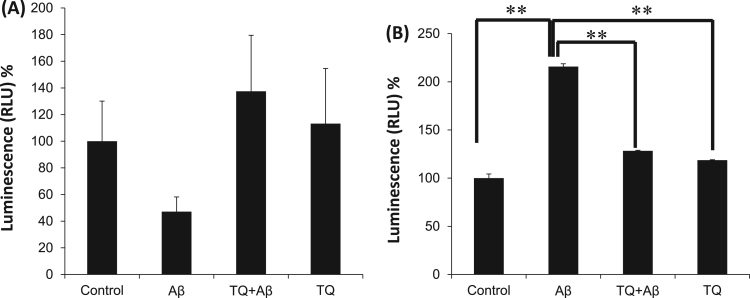
Effect of TQ and Aβ_1–42_ on Oxidative stress in hiPSC-derived cholinergic neurons. (A) The antioxidant GSH level was 53% decreased after treatment with Aβ_1–42_ (5 μM) comparing to control group. TQ co-treatment at (100 nM) protected GSH levels with 37% increase compared to control group. GSH level was assessed by the GSH-Glo™ Glutathione assay, n = 5. No significant difference was found in the effect between Control and Aβ (P = 1), Aβ and TQ+Aβ (P = 0.91) or Aβ and TQ (P = 0.45). (B) Intracellular ROS levels (H_2_O_2_) were significantly increased in Aβ_1–42_ (5 μM) treated group compared to control group. TQ (100 nM) co-treatment has attenuated the increase in ROS levels significantly as compared to Aβ_1–42_ treated group. Cultures were treated for 48 h and intracellular ROS was assessed by ROS-Glo™ H_2_O_2_ assay. (**P < 0.01: the group exposed to Aβ_1–42_ alone vs. control, vs. TQ + Aβ_1–42_ group, vs. TQ alone), n = 3. Values shown are the mean percent luminescence (where 100% = luminescence in control hiPSC-derived cholinergic neurons), ± SEM. Data were analyzed by one-way ANOVA, followed by the Holm-Bonferroni method.

In Fig. 2A, Aβ_1–42_ (5 μM) treatment induced a 53% decrease in GSH level as compared to control group with no significant difference (P = 1). Co-treatment with TQ (100 nM) has abolished Aβ_1–42_ effect and protected GSH level with a 37.5% increase in GSH level compared to control with no significant difference (P = 0.91).

### 3.4 Effect of Aβ_1–42_ and TQ on intracellular ROS level

To clarify the possible antioxidant effect of TQ, the accumulation of ROS was evaluated. In Fig. 2B, the treatment with Aβ_1–42_ (5 μM) caused a significant increase of 115% in H_2_O_2_ concentration compared to control (**P < 0.01). When the cells were treated with both Aβ_1–42_ and TQ, H_2_O_2_ concentration was decreased significantly with 87% decline compared to Aβ_1–42_ treated group (**P < 0.01).

### 3.5 Effect of Aβ_1–42_ and TQ on synaptic vesicles recycling

**Fig. 3:**
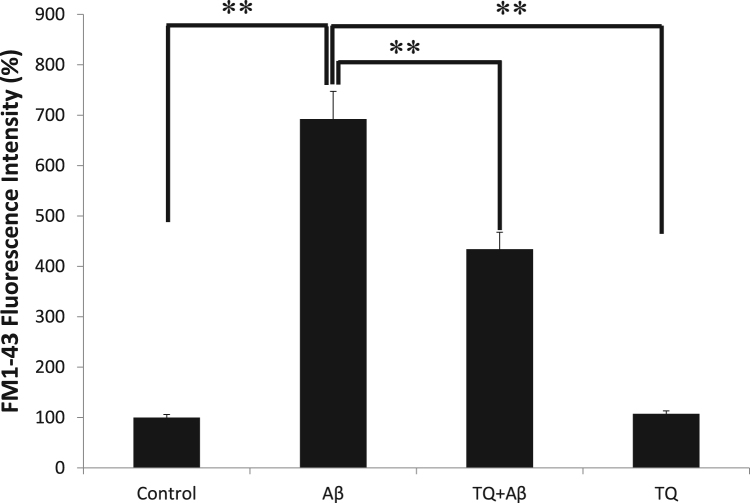
Effect of Aβ_1–42_ and TQ on synaptic activity in hiPSC-derived cholinergic neurons. Group treated with Aβ_1–42_ (5 μM) had a significant 6-fold increase in FM1–43 fluorescence intensity. TQ (100 nM) co-treatment reduced the increase in FM1–43 fluorescence intensity by 2.6-fold compared to Aβ_1–42_ treated sample. (**P < 0.01: the group exposed to Aβ_1–42_ alone vs. control, vs. TQ + Aβ_1–42_ group, vs. TQ alone) n = 5. Values shown are the mean percent fluorescence (where 100% = fluorescence in control hiPSC-derived cholinergic neurons), ± SEM. Data were analyzed by one-way ANOVA, followed by the Holm-Bonferroni method.

In Fig. 3, we demonstrated that the addition of Aβ_1–42_ (5 μM) for 48 h induced a 6-fold increase in the uptake of FM1–43 (**P < 0.01), which correlates with an increase in the synaptic activity. However, the co-treatment with TQ (100 nM) reduced the increase in the activity by 2.6-fold compared to Aβ_1–42_ treated sample (**P < 0.01).

## 4 Discussion

The major neuropathological hallmark of AD is the neurotoxic production and deposition of Aβ [25]. It has been demonstrated by numerous reports that oxidative stress plays an essential part in neuronal toxicity mediated by Aβ which triggers and facilitates neurodegeneration via a large extent of molecular actions that ultimately leads to the death of neurons [26]. Even though the precise mechanisms of Aβ cytotoxicity is still not completely elucidated, lots of studies tried to find how to inhibit the Aβ toxicity on the neurons [27].

TQ, a major active ingredient present in *Nigella sativa* seed's oil, has been subjected to a wide range of pharmacological investigations in recent years [28]. Due to its strong antioxidant capabilities, TQ has been demonstrated to protect the brain and the spinal cord from oxidative damage generated by different pathologies induced by a variety of free radicals [29], [30]. Moreover, TQ prevented cell death in rat cerebellar granule neurons and attenuated intracellular oxidative stress induced by Aβ in PC12 cells [31], [32]. Additionally, TQ was found to effectively ameliorate neurodegeneration [33], [34], [35].

Initial cognitive decline observed in AD is strongly related to the cholinergic basal forebrain dysfunction, which forms the basis of the ‘cholinergic hypothesis' of AD [36]. hiPSC-derived neurons are a great promising tool for they can be applied in human disease modeling, drug discovery, and cell transplantation [37], [38]. Accordingly, in the present study, the effect of TQ against Aβ_1–42_-induced neurotoxicity was investigated using hiPSC-derived cholinergic neurons.

When Aβ_1–42_ was applied to the cells, it resulted in decreased viability of hiPSC-derived cholinergic neurons marked by low levels of ATP, which was restored by the co-administration of TQ. In AD, low levels of ATP may lead to leakage of electrons and increase ROS production in the mitochondria, thereby leading to an additional source of oxidative stress [39]. It is well demonstrated that in neurodegenerative disorders, overproduction of ROS is implicated in neuronal apoptosis mediated by accumulation of Aβ [40], [41]. Additionally, it was reported that in the brains of AD patients and in neuronal cultures exposed to Aβ_1–42_, the dying cells exhibit the characteristics of apoptosis [42]. Particularly caspase 3 was reported to play an essential role in the execution phase of apoptosis induced by Aβ [43]. In our study, an increase in caspase 3/7 activities induced by Aβ_1–42_ was observed. However, TQ was able to suppress the activity of caspase 3/7 and hence apoptosis to the control sample level. Additionally, decreased GSH activity overtime results in H_2_O_2_ and lipid peroxidation accumulation, which leads to the characteristic pathological alterations of AD [44]. Therefore, an effective method to support the brain defense system should be performed by boosting antioxidants particularly GSH and associated enzymes. In this study, treatment of hiPSC-derived cholinergic neurons with Aβ_1–42_ induced a major reduction in GSH content and induced an increase in H_2_O_2_ generation. Intriguingly, the co-treatment with TQ restored the content of GSH and significantly inhibited the apparent increase in H_2_O_2_.

Since AD atrophy results from the degeneration of synapses [45], in our final experiment, the results indicated that Aβ_1–42_ caused a significant increase (6 fold) in the uptake of the fluorescent dye FM1–43 and therefore induced an increase in synaptic activity. When TQ was co-administrated there was a decrease in the synaptic activity by 2.6-fold compared to Aβ_1–42_ treated samples. In support of our findings, several previous studies have shown that at the presynaptic active zone, the increased number of available synaptic vesicles could be Aβ-dependent [46], [47]. Moreover, in AD, some of the important metal ions such as Zinc and Copper which are essential to regulate the neuronal activity in the synapses, suffer from disturbed homeostasis that could lead to increase in concentrations that can reach up to three times the normal levels observed in healthy brains [48]. Furthermore, Aβ_1–42_ may have the notable ability to enhance the mediated excitotoxicity of glutamate by specifically acting upon N-Methyl-D-aspartic acid (NMDA) receptors and thus, through an increased influx of Ca^2+^ [49], [50]. Previously, we demonstrated that TQ can protect hiPSC-derived neurons against alpha synuclein (αSN) induced synapse damage [51]. It has been demonstrated in several studies that small and soluble oligomers of Aβ are more toxic than its large fibrils [52], [53]. We have previously demonstrated that TQ was able to protect rat primary neurons against a range of Aβ_1–42_ induced toxicities possibly by inhibiting Aβ_1–42_ aggregation [23]. Moreover, a study demonstrated that TQ might be an effective Aβ inhibitor [54]. Although the exact mechanism of TQ is still not fully known, our findings demonstrate that the protective effects of TQ on neurons could be due to its ability to disaggregate Aβ accumulation, suppress its neurotoxic effect and consequently protect neuronal cells from Aβ induced neurotoxicity. In conclusion, to the extent of our knowledge, we believe that this work is the first to indicate the neuroprotective effect of TQ against Aβ_1–42_ induced neurotoxicity in cultured hiPSC-derived cholinergic neurons. The results strongly suggest the intracellular pathway of TQ to protect against the Aβ-induced toxicity on neurons of the central nervous system.
